# A pan-cancer analysis of the clinical and genetic portraits of somatostatin receptor expressing tumor as a potential target of peptide receptor imaging and therapy

**DOI:** 10.1186/s13550-020-00632-2

**Published:** 2020-04-25

**Authors:** Hyunjong Lee, Minseok Suh, Hongyoon Choi, Seunggyun Ha, Jin Chul Paeng, Gi Jeong Cheon, Keon Wook Kang, Dong Soo Lee

**Affiliations:** 1 Department of Nuclear Medicine, Seoul National University Hospital, Seoul National University College of Medicine, 28 Yongon-Dong, Jongno-Gu, Seoul, 110-744 Republic of Korea; 2 Division of Nuclear Medicine, Department of Radiology, College of Medicine, Seoul St. Mary’s Hospital, The Catholic University of Korea, Seoul, Republic of Korea; 3 Cancer Research Institute, Seoul National University, Seoul, Republic of Korea; 4 Department of Molecular Medicine and Biopharmaceutical Sciences, Graduate School of Convergence Science and Technology, Seoul National University, Seoul, Republic of Korea

**Keywords:** Somatostatin receptor, TCGA, Peptide receptor radiotherapy, Neuroendocrine tumor, Pan-cancer profile

## Abstract

**Purpose:**

Although somatostatin receptor (SST) is a promising theranostic target and is widely expressed in tumors of various organs, the indication for therapies targeting SST is limited to typical gastroenteropancreatic neuroendocrine tumors (NETs). Thus, broadening the scope of the current clinical application of peptide receptor radiotherapy (PRRT) can be supported by a better understanding of the landscape of SST-expressing tumors.

**Methods:**

SST expression levels were assessed in data from The Cancer Genome Atlas across 10,701 subjects representing 32 cancer types. As the major target of PRRT is SST subtype 2 (SST2), correlation analyses between the pan-cancer profiles, including clinical and genetic features, and SST2 level were conducted. The median SST2 expression level of pheochromocytoma and paraganglioma (PCPG) samples was used as the threshold to define “high-SST2 tumors.” The prognostic value of SST2 in each cancer subtype was evaluated by using Cox proportional regression analysis.

**Results:**

We constructed a resource of SST expression patterns associated with clinicopathologic features and genomic alterations. It provides an interactive tool to analyze SST expression patterns in various cancer types. As a result, eight of the 31 cancer subtypes other than PCPG had more than 5% of tumors with high-SST2 expression. Low-grade glioma (LGG) showed the highest proportion of high-SST2 tumors, followed by breast invasive carcinoma (BRCA). LGG showed different SST2 levels according to tumor grade and histology. IDH1 mutation was significantly associated with high-SST2 status. In BRCA, the SST2 level was different according to the hormone receptor status. High-SST2 status was significantly associated with good prognosis in LGG patients. High-SST2 status showed a trend for association with poor prognosis in triple-negative breast cancer subjects.

**Conclusion:**

A broad range of SST2 expression was observed across diverse cancer subtypes. The SST2 expression level showed a significant association with genomic and clinical aspects across cancers, especially in LGG and BRCA. These findings extend our knowledge base to diversify the indications for PRRT as well as SST imaging.

## Introduction

Somatostatin receptor (SST) is one of the most representative imageable targets for not only diagnosis but also therapy. In particular, several radioligands targeting SST have been developed, such as ^68^Ga-DOTATOC and ^68^Ga-DOTATATE, which are now commonly used in the diagnosis of neuroendocrine tumors (NETs) via positron emission tomography (PET). Because of the specific binding to SST of these radiopharmaceuticals and the overexpression of SST in NETs, SST imaging via PET has high sensitivity and specificity for detecting tumor lesions. Furthermore, beta- or alpha-emitting radiopharmaceuticals can be used as peptide receptor radiotherapy (PRRT) agents to destroy tumor tissues. The therapeutic effect of PRRT is stronger than that of conventional octreotide treatment for NETs [1–4]. Nevertheless, the current application of these radiopharmaceuticals is limited to SST-positive gastroenteropancreatic NETs so far, while unresectable cases of metastatic NETs are not uncommon [5].

Even though SST is a key molecule of NETs, it is not exclusively expressed in NETs alone. There have been a few reports about incidental DOTATOC or DOTATATE uptake in tumors other than NETs [6–8]. In particular, the somatostatin analogue is known to show remarkably increased uptake in meningioma [9]. This implies the possibility of clinical application of SST targeting in other tumors beyond NETs. Furthermore, SST-expressing tumors can have different biological characteristics than tumors without SST expression, even within the same tumor type, as SST expression suggests neuroendocrine differentiation of tumors [10, 11]. In this regard, some reports have suggested an association between clinical outcome and the expression of SST in glioma, thyroid cancer, and lung cancer [12–14]. Thus, broadening the scope of the current clinical application of SST targeting may include precise diagnostics, such as subtyping of tumors and risk stratification of various types of cancer other than NETs, as SST imaging noninvasively provides information on tumor extent and metastasis in the whole body as well as the status of SST expression.

The Cancer Genome Atlas (TCGA) project is a worldwide database that provides comprehensive data including genetic, histopathologic, and clinical information for various cancer types [15]. Using these open access data, we can conduct diverse and creative analyses in a large number of subjects for topics that are difficult to assess in current clinical settings. To date, no previous study has comprehensively evaluated the landscape of SST expression throughout various cancer subtypes other than NETs. Here, we developed an interactive resource to comprehensively analyze SST expression across cancers using TCGA data associated with genomic alterations, clinical features, and prognosis (https://choih.shinyapps.io/sstr/).

## Method

### Data sources and preprocessing

All data were obtained from TCGA projects. Using the “recount2” R package, we downloaded the gene expression data of 11,284 subjects representing 32 cancer types [16]. The “BiocManager install” function was used to download the “recount2” R package. Raw sequencing data were scaled to the total number of mapped reads and read lengths. Subsequently, log2 normalization was performed. A total of 10,699 subjects with primary tumor tissue and/or normal solid tissue were selected from all the subjects; the other 585 subjects were excluded. We excluded metastatic tumors because the TCGA project aimed to analyze primary tumor lesions, and SST expression can be different according to not only cancer subtype but also metastatic organ. The gene expression level of all types of SST (SST1, SST2, SST3, SST4, and SST5) was calculated in primary tumor tissue and normal solid tissues. Using the “TCGAmutations” R package, we downloaded precompiled somatic mutation data from the TCGA project [17]. Clinical information, including histopathological findings and survival data, was downloaded from the Cancer Genomic Data Server using the “cgdsr” R package. Somatic mutation data and clinical information data were merged with gene expression data in identical subjects. All abbreviations for cancer subtypes and gene names are defined in the supplementary material.

### Statistical analysis

First, SST expression in normal tissue and tumor tissue was compared across cancers using the Mann-Whitney test. Among all SSTs, we focused on SST2, which is a representative SST molecule used for SST-targeted imaging and therapy. Pheochromocytoma and paraganglioma (PCPG) samples were selected as reference tissues to define high-SST2 tumors. Normal kidney tissue samples were also selected as another reference tissue. The median SST2 expression in PCPG or normal kidney tissue samples was defined as the threshold to classify high-SST2 tumors and low-SST2 tumors. The proportion of high-SST2 tumors across cancers was calculated.

To explore the significant genomic alterations in high-SST2 tumors, two cohorts were constructed based on the SST2 expression level. Using the “maftools” R package, gene mutation profiles of high-SST2 tumors and low-SST2 tumors were compared by Fisher’s exact test [18].

For survival analyses, the prognosis of patients with high-SST2 tumors and low-SST2 tumors was compared using Cox proportional regression analysis for each cancer subtype. For additional analysis, the prognosis of patients with different histopathological types was compared in the same cancer subtype. All statistical analyses were performed by the R software (v 3.6.1).

## Results

### SST expression across cancers

**Fig. 1:**
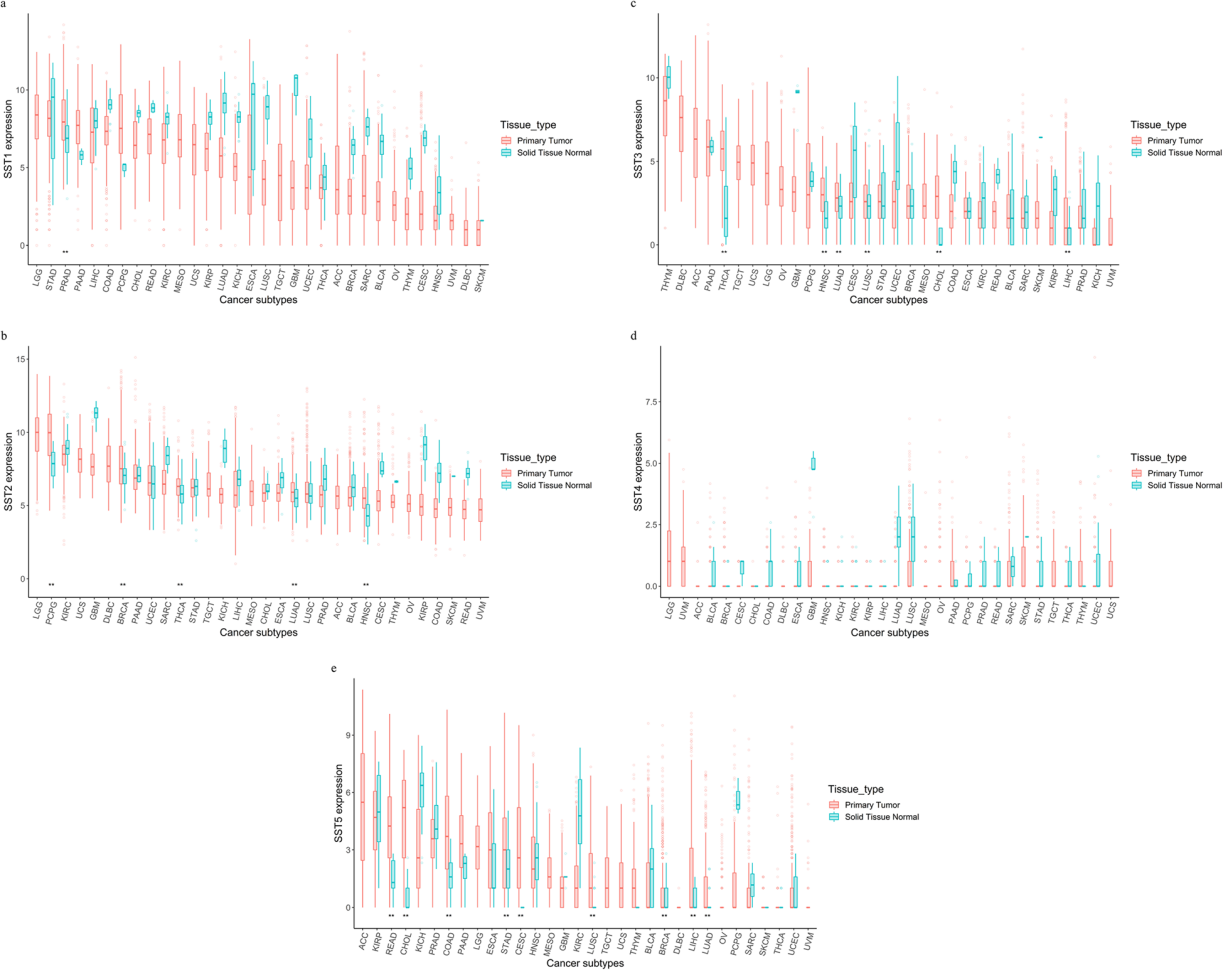
SST expression level of primary tumor tissue and normal solid tissue in the pan-cancer. Two asterisks (**) mean statistical significance, *p* value lesser than 0.05. **a** SST1 expression. **b** SST2 expression level in primary tumor tissue was revealed higher than that in normal solid tissue in five cancer subtypes: PCPG, BRCA, THCA, LUAD, HNSC. Median value of SST2 expression was the highest in LGG and lowest in UVM. **c** SST3 expression. **d** SST4 expression. **e** SST5 expression

There were 9960 primary tumor and 739 normal tissue samples (Table 1). The SST1 expression level in primary tumor tissues was higher than that in normal tissues in prostate adenocarcinoma (PRAD). The SST2 expression level in primary tumor tissues was higher than that in normal tissues in the following cancer subtypes: PCPG, breast invasive carcinoma (BRCA), thyroid carcinoma (THCA), lung adenocarcinoma (LUAD), and head and neck squamous cell carcinoma (HNSC). The SST3 expression level in primary tumor tissues was higher than that in normal tissues in the following cancer subtypes: THCA, HNSC, LUAD, lung squamous cell carcinoma (LUSC), cholangiocarcinoma (CHOL), and liver hepatocellular carcinoma (LIHC). The SST4 expression level in primary tumor tissues showed no significant difference from that in normal tissues in all cancer subtypes. The SST5 expression level in primary tumor tissues was higher than that in normal tissues in the following cancer subtypes: rectal adenocarcinoma (READ), CHOL, colon adenocarcinoma (COAD), stomach adenocarcinoma (STAD), cervical and endocervical cancer (CESC), LUSC, BRCA, LIHC, and LUAD (Fig. 1a–e).

**Table 1:** Numbers of primary tumor and normal tissue samples

**Cancer subtypes**	**ACC**	**BLCA**	**BRCA**	**CESC**	**CHOL**	**COAD**	**DLBC**	**ESCA**	**GBM**	**HNSC**	**KICH**	**KIRC**	**KIRP**	**LGG**	**LIHC**	**LUAD**
	79	414	1127	304	36	503	48	184	157	502	66	543	290	514	371	540
	0	19	112	3	9	41	0	13	5	44	25	72	32	0	50	59
**Cancer subtypes**	**LUSC**	**MESO**	**OV**	**PAAD**	**PCPG**	**PRAD**	**READ**	**SARC**	**SKCM**	**STAD**	**TGCT**	**THCA**	**THYM**	**UCEC**	**UCS**	**UVM**
	504	87	422	178	179	505	166	259	103	415	150	505	120	553	57	80
	51	0	0	4	3	52	10	2	1	37	0	59	2	35	0	0

**Fig. 2:**
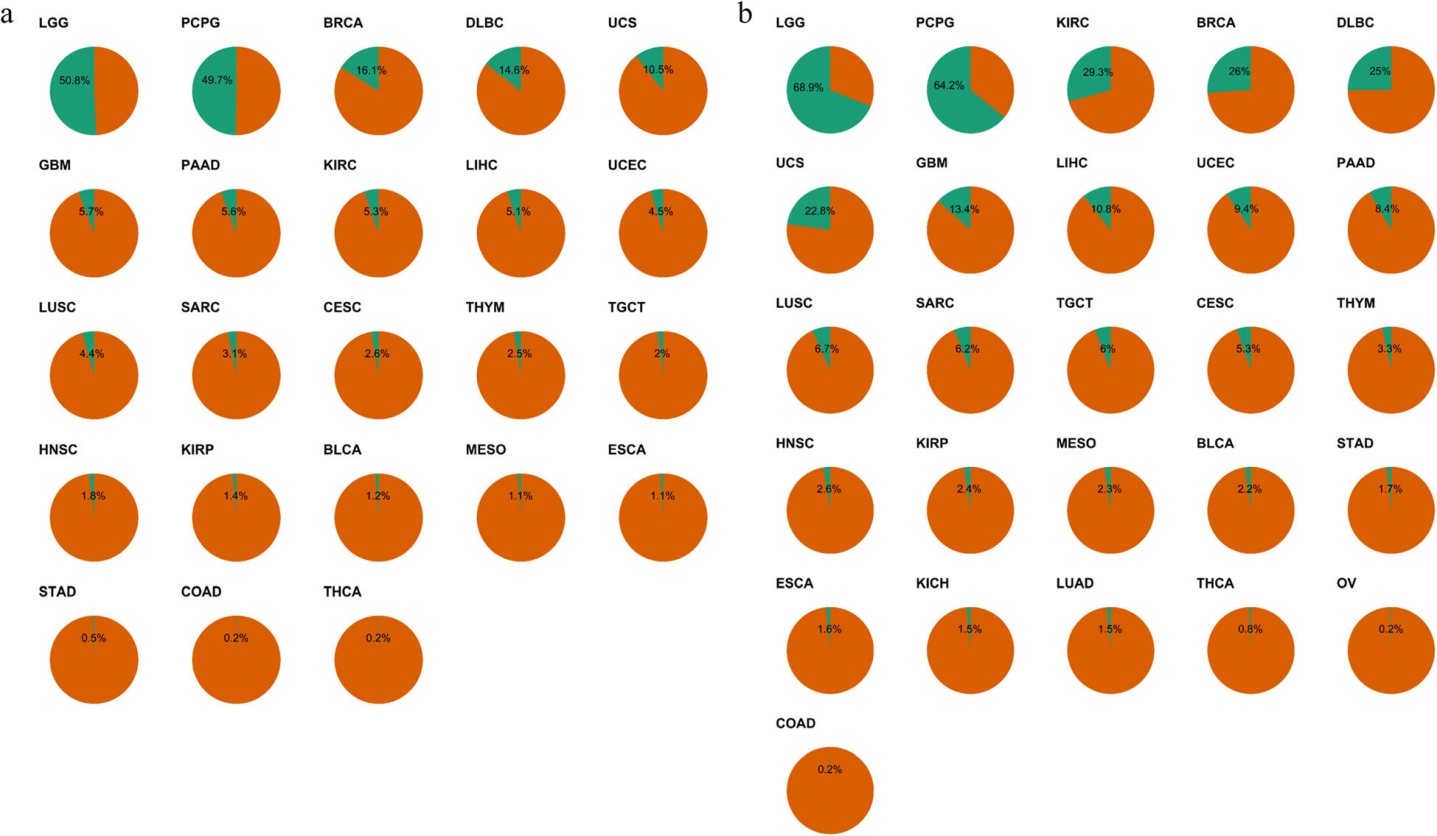
Proportion of high-SST2 tumor in the pan-cancer. Green color represents a proportion of high-SST2 tumors of each tumor subtype based on the expression level of PCPG as a reference value (**a**) and the expression level of normal kidney tissue as a reference value (**b**)

Based on the expression level of SST2 in PCPG as a reference value, eight of the 31 cancer subtypes other than PCPG had more than 5% of tumors with high-SST2 expression, and 19 of them had more than 1% of tumors with high-SST2 expression. LGG showed the highest proportion (50.8%) of high-SST tumors, followed by BRCA (16.1%) (Fig. 2a). Based on the expression level in normal kidney tissue as a reference value, fourteen of the 32 cancer subtypes had more than 5% of tumors with high-SST2 expression, and 23 of them had more than 1% of tumors with high-SST2 expression. Low-grade glioma (LGG) showed the highest proportion (68.9%) of high-SST tumors, followed by PCPG (64.2%) and kidney renal clear cell carcinoma (KIRC) (29.3%) (Fig. 2b).

### Association of SST2 with genomic alterations

**Fig. 3:**
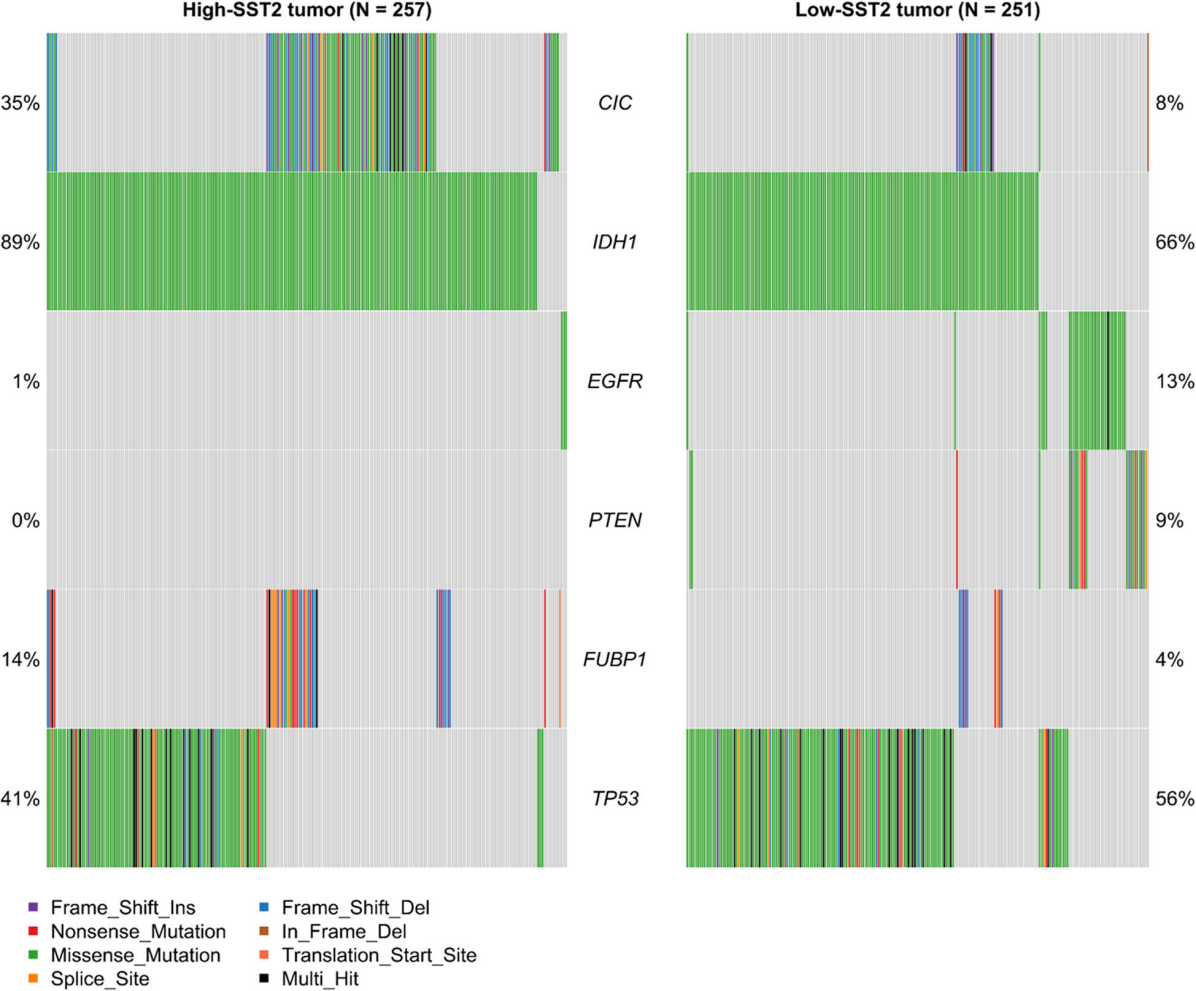
Gene mutational profiles in the high-SST2 LGG and low-SST2 LGG. Six genes showed genetic alteration in accordance with SST2 status. IDH, CIC, and FUBP1 mutations were more in high-SST2 tumor. On the contrary, EGFR, PTEN, and TP53 mutations were more in low-SST2 tumor

We investigated whether SST2-expressing tumors were associated with genomic alterations in several tumor types. In this analysis, the expression level of SST2 in PCPG was defined as the reference value. Mutational profiles were significantly associated with high-SST2 status in LGG, which showed the highest proportion of high-SST tumors, including isocitrate dehydrogenase 1 (IDH1), capicua transcriptional repressor (CIC), and far upstream element binding protein 1 (FUBP1) mutations. In contrast, epidermal growth factor receptor (EGFR), phosphatase and tensin homolog (PTEN), and tumor protein 53 (TP53) mutations were more common in low-SST2 tumors than in high-SST2 tumors (Fig. 3). In pancreatic adenocarcinoma (PAAD), Kirsten rat sarcoma (KRAS) and TP53 mutations were associated with low-SST2 status. In uterine corpus endometrial carcinoma (UCEC), catenin beta 1 (CTNNB1) mutation showed an association with low-SST2 status. When high-SST2 tumors were defined with the expression level in PCPG as a reference value, SST2-based tumor subtypes were not significantly associated with gene alterations in other cancer subtypes, including BRCA, PCPG, and KIRC. The associated genomic profiles according to SST expression can be explored at https://choih.shinyapps.io/sstr/.

### Association of SST2 with histopathologic findings and prognosis

**Fig. 4:**
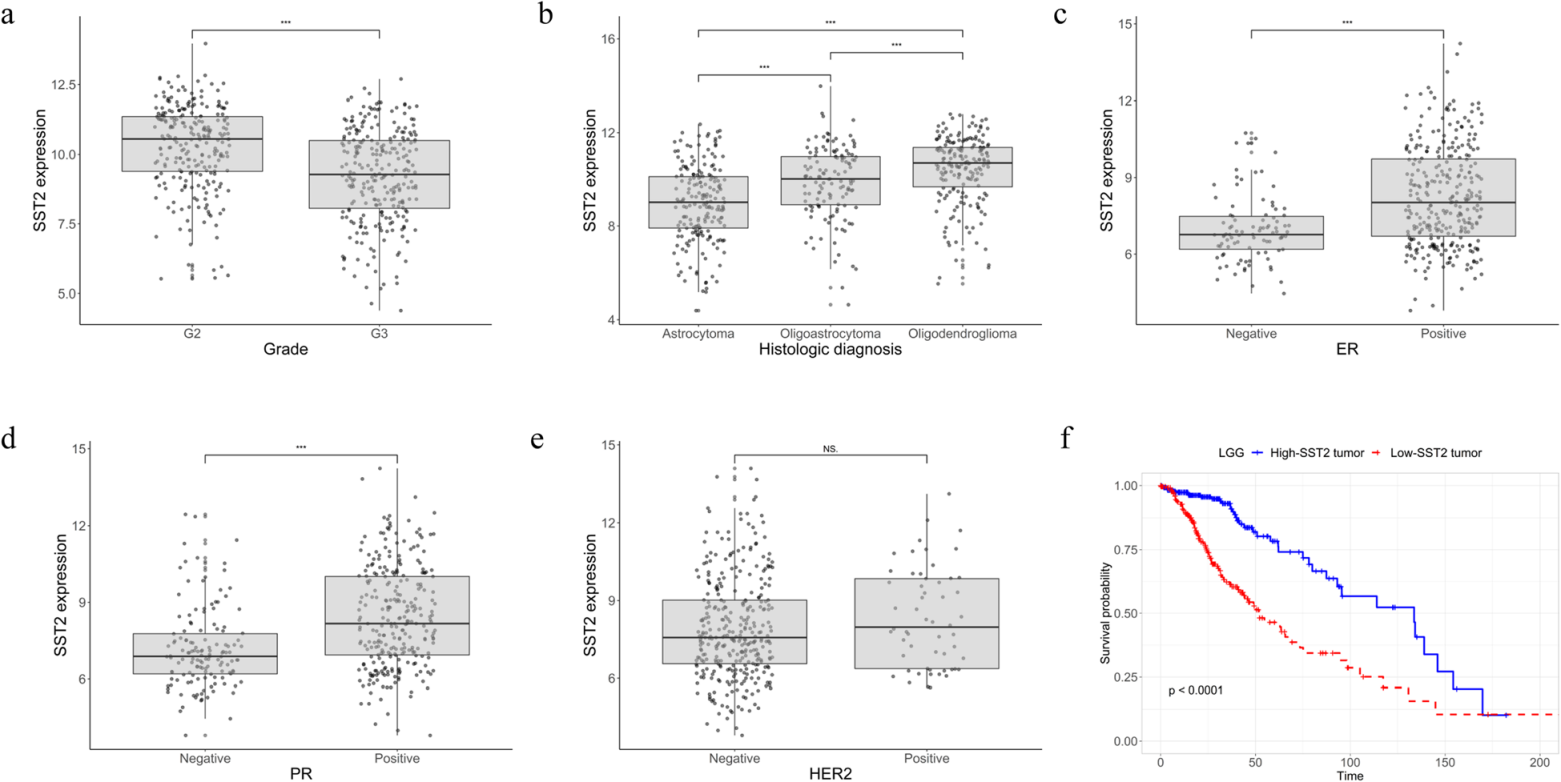
Association of SST2 with histopathologic findings and prognosis in LGG and BRCA. Three asterisks (***) mean *p* value lesser than 0.001. NS means no statistical significance (*p* > 0.05). SST2 expression level showed significant difference in accordance with histological grade (**a**) and type (**b**) of LGG. SST2 expression level showed significant difference in accordance with hormone receptor status of BRCA (**c**, **d**). HER2 expression status showed no association with SST2 expression level (**e**). High-SST2 status was significantly associated with a good prognosis in LGG (*p* < 0.0001) (**f**)

In LGG, SST2 expression was significantly different according to tumor grade and histology. The SST2 level in G2 grade tumors was significantly higher than that in G3 grade tumors (Fig. 4a). In terms of histologic subtypes of LGG, oligodendroglioma showed the highest SST2 level, followed by oligoastrocytoma and astrocytoma, with statistical significance (Fig. 4b). In BRCA, the SST2 level was different according to the hormone receptor status. The presence of estrogen receptor (ER) or progesterone receptor (PR) was correlated with high-SST2 expression. However, human epidermal growth factor receptor 2 (HER2) expression was not correlated with the SST2 expression level (Fig. 4c–e). High-SST2 status was significantly associated with a good prognosis in LGG (Fig. 4f). No significant prognostic impact of high-SST2 status was shown in breast cancer patients overall. Additionally, there was no prognostic impact of high-SST2 status in ER-positive and PR-positive breast cancer patients. However, high-SST2 status tended to be a poor prognostic factor in triple-negative breast cancer (TNBC) subjects specifically, but the relationship was not statistically significant. In addition, SST2 status showed prognostic impact in thymoma and glioblastoma when the level of expression in normal kidney tissue was defined as the reference (Supplementary fig. 1).

## Discussion

In this study, we developed a resource to explore the landscape of SST expression across tumor profiles, and we screened SST2 expression levels across cancers. Tumor tissues showed higher SST2 expression, the major target of DOTATOC and DOTATATE PET, than normal tissues not only in PCPG but also in BRCA, THCA, LUAD, and HNSC. These results imply the potential use of SST-targeted imaging in various tumors other than NETs to demonstrate uptake in tumors compared to normal tissue.

We explored the SST2 expression level across cancers based on the expression in PCPG as a cutoff, and PCPG is well known to show high-SST2 expression. Eight of the cancer subtypes analyzed had more than 5% of tumors with high-SST2 expression with PCPG levels as a reference. Additionally, we revealed that fourteen of the 32 cancer subtypes had more than 5% of tumors with high-SST2 expression when SST2 expression in normal kidney tissue was used as the reference. Normal kidney tissue shows relatively higher SST2 expression than other normal tissues, as shown in Fig. 1. This result corresponds with the findings of a previous analysis of human tissue-specific expression [19]. Taken together, these results suggest that a wide range of tumors may show enough uptake of SST-targeting molecules to allow theranostic approaches to be used in diseases beyond NETs and PCPG [5, 20, 21].

There are several significant results from this study that closely mirror findings from existing studies about the application of somatostatin analogues in various tumors. First, the present study is in perfect accord with previous knowledge that gliomas can express SST2 [12, 22, 23]. The results of this study support previous attempts of somatostatin targeting in glioma [24, 25]. Second, thyroid cancer has shown somatostatin analogue uptake in a few reports [26, 27]. In contrast, the present study found that there was a low proportion of high-SST2 tumors in THCA. This difference seems to be due to the nature of the tumor. Because previous studies mainly focused on iodine therapy in refractory thyroid cancer, the proportion of well-differentiated thyroid cancers was relatively small. Third, from our results, we can consider utilization of SST2 targeting for cancer subtypes such as BRCA on a pilot basis. This corresponds with a previous report describing the incidental uptake of DOTATOC in breast cancer [6].

Several gene mutations were noted to have a correlation with SST2 expression in LGG. In particular, the IDH1 mutation was revealed to be associated with SST2 expression, which is supported by a previous study [12]. The close association between IDH1 mutation and SST2 expression was also verified by the association of high-SST2 with good prognosis in LGG. The presence of IDH1 mutation is the most common factor used to classify tumor subtypes in terms of disparate molecular pathogenesis and favorable prognosis [28–30]. Additionally, not only IDH1 but also CIC and FUBP1 mutations demonstrated a positive association with SST2 expression. This finding is also consistent with previous studies that showed an association between IDH1, CIC, and FUBP1 mutations and a favorable prognosis [31]. In contrast, EGFR, PTEN, and TP53 mutations showed a negative association with SST2 expression. This corresponds with previous knowledge that these mutations are poor prognostic factors [32–34]. This association could lead to the future application of SST-targeted imaging as a noninvasive biomarker to noninvasively evaluate glioma subtypes to predict prognosis and plan treatment.

LGG and BRCA are the most representative cancer subtypes expressing SST2 other than NETs. Therefore, more details about the relationship between clinico-histological characteristics and SST2 expression, especially in those cancer subtypes, are needed. In LGG, grade 2 tumors and oligodendrogliomas showed higher SST2 expression levels than tumors of other grades and histologies, consistent with a previous report [35]. Considering the association between gene mutations and SST2 expression, a high level of SST2 expression can be deemed a strong alternative to favorable prognostic markers, such as IDH1 mutation or tumor grade. Not surprisingly, high-SST2 status in LGG was significantly associated with a good prognosis in the present study. Notably, SST2 expression was also high in normal brain tissues (as represented by normal tissues from GBM) (Fig. 1b). Nonetheless, in terms of theranostic targeting, normal brain uptake is minimal because of the blood-brain barrier. In this regard, as tumor accumulation of the SST2-targeting agents is also affected by the disruption of the blood-brain barrier, noninvasive assessment of the SST2 status in LGG requires further careful kinetic studies. In BRCA, high-SST2 expression showed a correlation with the presence of hormone receptors. This strongly supports a previous study demonstrating an association between SST2 expression and hormone receptor expression by histopathologic findings [36]. Although hormone receptors are favorable prognostic factors in breast cancer patients, SST2 showed no significant prognostic power in this study. Despite the lack of relationship with prognosis, the association of hormone receptor status with SST2 expression might enable the use of SST-targeted imaging to noninvasively characterize metastatic breast cancer lesions to determine the potential intertumoral heterogeneity in terms of hormone receptor status [37].

Taken together, these results show that high-SST2 status correlates well with well-known key biomarkers of LGG and BRCA. In this regard, SST2-targeted imaging and therapies have two clinical applications. First, DOTATOC or DOTATATE PET may be a powerful tool to screen patients with a good prognosis. To the best of our knowledge, no clinical study has explored the clinical impact of somatostatin analogue imaging in LGG and BRCA. Moreover, a key advantage of PET imaging is that it is noninvasive whole-body imaging that enables the evaluation of multiple metastatic tumor lesions with consideration of tumor heterogeneity. Thus, it could provide spatial and temporal dynamics of surrogate biomarkers to characterize the current status of each tumor lesion for precision oncology [38]. Second, therapeutic radiopharmaceuticals, including Lu-177-labeled DOTATATE, may be a feasible option for high-SST2 tumors regardless of the organ-based subtypes. Despite IDH1 mutations and hormone receptor expression being good prognostic factors, recurrence of brain tumor patients with IDH1 mutations and breast cancer patients with hormone receptor expression is frequently observed in the clinical setting. If we broaden the indications for PRRT, which is a powerfully selective therapeutic molecule in other cancer subtypes, more cancer patients could benefit. In terms of a companion diagnostics for PRRT, tumors with high radiotracer avidity are expected to have a good response due to their higher radiation dose regardless of the primary tumor site. Therefore, the indications for SST2-targeted PRRT could be extended to such imaging biomarker-based treatment, which might be supported by basket trials [39]. Although high-SST2 tumors are present in small proportions in various cancer types, the identification of DOTATOC- or DOTATATE-avid tumors combined with conventional treatment may result in good results. Further clinical validation based on specially designed basket trials is warranted to realize this broad-range theranostic approach. Notably, there was high-SST2 expression in some TNBC patients. TNBC is well known to have more resistance to chemotherapy, so the cure rate is relatively lower than that in hormone receptor-positive breast cancer. This implies that SST-targeting radiotherapeutics may have potential feasibility as alternative and adjuvant therapeutic agents for TNBC patients.

There is a limitation in this study. Occasionally, the protein expression level may differ from RNA expression. Nonetheless, RNA expression grossly reflects the protein level and function of the molecule. Since there is not a large protein-level database of multiple cancer subtypes, we included only RNA expression for the present study. Further study can be performed to investigate SST expression across cancers using large-scale data on protein expression.

## Conclusion

The associations of SST expression with genomic alterations, clinical features, and prognosis across cancers can be explored by our web-based resource. Higher SST2 expression levels were found in various tumor subtypes, especially in LGG and BRCA. Several gene mutations and histopathological findings, which are known to be beneficial prognostic markers, were associated with high-SST2 expression. High-SST2 status showed a positive correlation with clinical outcome in LGG but tended to be negatively correlated with clinical outcome in TNBC. These results suggest the potential of SST2-targeted imaging and therapies in a wide range of tumors beyond the current limited indication, well-differentiated neuroendocrine tumors.

## Supplementary information

**Additional file 1:.** Supplementary fig. 1 Association between prognosis and SST2 expression level. High-SST2 status tended to be a poor prognostic factor when limited to TNBC subjects without statistical significance (p = 0.16) (a). When normal kidney tissue was defined as a reference, thymoma with low SST2 status and glioblastoma with high-SST2 status showed good prognosis (b-c)

## Data Availability

The clinical data can be found at the GDC portal (https://portal.gdc.cancer.gov/). The mutation data can be downloaded from the R library, “TCGAmutations” (https://github.com/PoisonAlien/TCGAmutations). Our processed results are available at a web-based resource (https://choih.shinyapps.io/sstr/). Software and resources used for the analyses are described in each method section.
