# Supplementary figures and images for: A pan-cancer analysis of the clinical and genetic portraits of somatostatin receptor expressing tumor as a potential target of peptide receptor imaging and therapy
Source: EJNMMI Res. 2020 Apr 25;10:42. doi: 10.1186/s13550-020-00632-2 (PMC7183516; doi:10.1186/s13550-020-00632-2)

Supplementary fig. 1

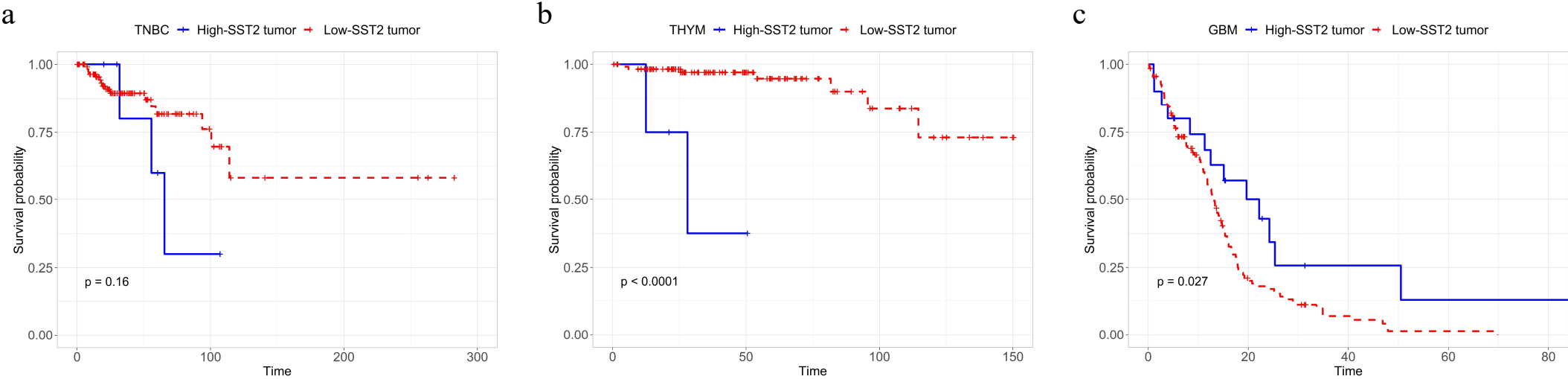

Supplement: Supplementary file 1 — Additional file 1:. Supplementary fig. 1 Association between prognosis and SST2 expression level. High-SST2 status tended to be a poor prognostic factor when limited to TNBC subjects without statistical significance (p = 0.16) (a). When normal kidney tissue was defined as a reference, thymoma with low SST2 status and glioblastoma with high-SST2 status showed good prognosis (b-c) [file 13550_2020_632_MOESM1_ESM.pdf]
